# Supplementary material for: Chromothripsis during telomere crisis is independent of NHEJ, and consistent with a replicative origin
Source: Genome Res. 2019 May;29(5):737–49. doi: 10.1101/gr.240705.118 (PMC6499312; doi:10.1101/gr.240705.118)
Supplement: Supplemental Material [file supp_gr.240705.118_Supplemental_file_1.zip › contigs/annotated_contigs/DB79/contig.2.DB79_length_458_mean_cov_5.89519650655.docx]

**DB79_length_458_mean_cov_5.89519650655**

```
GGATTGTGATATTTTCCTGTTGAACTAGTCCATTTATCACTATATAATGTCCCTCTTTGTCTTTTTTAACTGCTATTGCTTTAAGGTTT
 >chr7:115717589-115717787 - E=1e-107 p=1e-03
TTTTTGTCTGATATAAGAATAACTACTCTTGCTTGCATTTGGTTTCCATTTGCATGGAATATCTTTTTTCACCCCTTTACCTTAAGTTT

ATATGAGTTCTTATGTG|TTA|CTTTGGATTTAATCTATCTCCTTTTTCTAATGTGTTAAGGTAGAAGCTGAAGTCTTGGACATGTGAT
                  >chr7:115698803-115699066 - E=9e-144
CTTTATTTTTTTAAATACAGGCATTTAGTACTAAAAATTCGTCCCTCAATATTGCTTTATCGGCATCTCACAAATTTAAGTCTGTTTTA

CTCCTGTCTTCATTCAGTTCAAAAATACTTTCTAATTTCCCTTTTTATTTTTTTGAACCATGGGTTATTTTAAAGTATATTATATTATT

TTGCTCTAAATATTT
```
